# Délire dermatozoïque: à propos d'un cas

**DOI:** 10.11604/pamj.2013.16.25.2895

**Published:** 2013-09-25

**Authors:** Masmoudi Jawaher, Feki Ines, Sallemi Rim, Baati Imen, Jaoua Abdellaziz

**Affiliations:** 1 Service de psychiatrie A, CHU Hédi Chaker, 3029 Sfax, Tunisie

**Keywords:** Délire dermatozoique, ekbom, dépression, dermatozoic delirium, ekbom, depression

## Abstract

Le délire dermatozoïque est un délire particulier survenant chez les personnes âgées et caractérisé par la présence d'un délire hallucinatoire essentiellement cénesthésique, avec la conviction d’être infesté par des petites bêtes. Ce délire aboutit à des lésions de grattage pouvant être graves, mais sans aucune étiologie organique, et leurrant souvent le dermatologue. Notre observation est originale, par le fait que le patient a été adressé par un ophtalmologue, et faisant étendre le champ du diagnostic à plusieurs spécialités, s'occupant des patients âgés. il s'agit d'un patient âgé de 67 ans, ayant présenté da façon concomitante à une intervention chirurgicale et au mariage de son dernier fils, un délire hallucinatoire avec une conviction d’être attaqué par des moustiques lui suçant le sang. Des lésions d'excoriation cutanée ont été secondaires à ce délire, et ont été à l'origine d'une consultation psychiatrique. L’évolution après six mois de traitement a été marquée par l'enkystement du délire et l'amélioration des lésions cutanées. Réputé rare, le délire dermatozoïque pourrait être déroutant si on ne fait pas le diagnostic positif. Tout clinicien peut être confronté dans sa pratique à ce type de syndrome. Sa compréhension facilite une prise en charge adéquate, basée essentiellement sur un soutien psychologique et un traitement neuroleptique.

## Introduction

*“Toute fonction psychique se développe par appui sur une fonction corporelle dont elle transpose le fonctionnement sur le plan mental”* [1]. Cette citation renvoie à plusieurs pathologies qui se trouvent à l'intersection de la psychiatrie et différentes pathologies à expression somatique. La peau, en particulier, entretient des relations privilégiées avec le psychisme, avec d'ailleurs une origine embryonnaire commune. Ces relations peuvent se trouver perturbées, notamment chez le sujet âgé, dont aussi bien le cerveau que la peau subissent les effets de la sénilité. C'est le cas du délire dermatozoïque ou syndrome d'Ekbom, qui correspond à un délire d'apparition tardive, sans incohérence, ni atteinte démentielle et dont l’élément spécifique est la conviction de la présence de parasites sous la peau. Nous rapportons le cas d'un patient, adressé en psychiatrie, et présentant un délire d'infestation cutané dont l'originalité réside dans le cours évolutif, soit l'apparition d'hallucinations initialement visuelles puis cénesthésiques l'ayant plutôt amené en ophtalmologie, malgré des lésions cutanées sévères. D'où l'intérêt d'actualiser les connaissances, pour sensibiliser les cliniciens, de toutes spécialités, à dépister, et traiter ce syndrome réputé comme rare, mais plutôt sous diagnostiqué [2–5].

## Patient et observation

Mr AB âgé de 67 ans, nous a été adressé par un ophtalmologue pour “délire hallucinatoire”: vision de moustiques qui le piquent, surtout le soir. Il est, en fait, suivi pour cataracte bilatérale, avec rétinopathie diabétique. Il est à la retraite depuis 7 ans, et vit seul avec sa femme depuis plus d'une année, après le “départ” de ses 5 enfants, tous mariés.

L'histoire des troubles remonte à trois mois, dans les suites d'une phacotomie, soit une durée d’évolution d'un an et demi. Elle a été marquée par l'installation progressive de troubles du comportement, soit une attitude d'autoprotection continue par une couverture, en rapport avec la vision de moustiques qu'un intrus dans la maison lui jette dessus. Ces moustiques l'auraient attaqué puis se seraient fixées sur son corps. Il ne cessait d'essayer de les enlever par un grattage incessant, avec des lésions cutanées sévères.

L'attitude initiale de l'ophtalmologue a été de rassurer le patient et de tenter de lui expliquer le caractère plutôt “imaginaire” de ses troubles, espérant, en vain, le convaincre de l'inexistence de ces insectes. Devant l'absence d'amélioration le patient nous a été adressé.

A l'examen, le patient était anxieux, triste, pessimiste et craignant que les moustiques lui sucent son sang. Sa personnalité a été marquée des traits paranoïaques, avec surtout une psychorigidité, et une méfiance. Il présentait un délire d'infestation cutanée, à mécanisme hallucinatoire, cénesthésique et visuel. L'examen psychiatrique a permis d’éliminer une symptomatologie démentielle (MMSE normal) et le scanner cérébral s'est révélé sans particularités. L'examen neurologique a été normal. Notre attitude a été de lui prescrire de façon simultanée un antidépresseur (fluoxétine), un anxiolytique (lorazépam) et un neuroleptique (halopéridol).

L’évolution, au bout de six mois, a été marquée par une amélioration de l'humeur, du sommeil et une réduction de la charge anxieuse, mais le patient a continué à se protéger par sa couverture. Cependant, il ne perturbait plus le fonctionnement familial, et les lésions de grattage se sont cicatrisées.

## Discussion

Les premières descriptions cliniques de cas de croyance d'infestation cutanée par des insectes remonte au IXXème siècle, avec des terminologies différentes, telles que “l'acarophobie” de Thibierge (1894), et la « nevrodermie parasitophobique » de Perrin (1896) [6]. Mais, C'est à K. Ekbom (1938) [7], que revient le mérite de la description princeps de ce syndrome. Le délire de Mr AB s'est installé dans les suites de son intervention.

Plusieurs auteurs signalent un rapport intime entre l'installation, insidieuse ou brutale, et un événement précis: contact avec un animal, de la saleté, une affection dermatologique, un bouleversement dans la vie du sujet, etc [2, 8]. Pour Mr AB il y a eu à la fois le départ des enfants et l'intervention chirurgicale. La concomitance de l'apparition du délire de Mr AB et du départ du dernier enfant rajoute un autre facteur de stress, soit l'accentuation de son isolement, puisqu'il est déjà retraité: plusieurs auteurs signalent l'isolement social comme facteur favorisant l’éclosion de ce syndrome [9].

L'adhésion au délire est totale. La réaction du patient à ce délire et à la sensation corollaire de démangeaisons, de brûlure ou de picotement, mène typiquement à un grattage obstiné et tenace, pour se soulager, et faire sortir les parasites et/ou les visualiser. Ce qui sera responsable d'excoriations ou de lésions surinfectées. On peut voir aussi des réactions de protection contre les parasites présumés: bains répétés, utilisation d'insecticides, etc [8]; attitude d'autoprotection concernant Mr AB. Le désir d'apporter des spécimens à une instance scientifique, médicale ou non, est également caractéristique. C'est le “matchbox sign” [10, 11]

Ces sensations, sont expliquées soit par des hallucinations cénesthésiques ou par l'interprétation erronée des sensations cutanées, qui semblent migrer sous la peau et donnent l'impression de déplacement d'un être vivant, dont la nature est en rapport avec le système socioculturel du patient: vers, microbes, acariens, poux, etc. La vision hallucinatoire de cet être renforce l'adhésion au délire. Pour notre patient ce sont les hallucinations visuelles qui ont précédé celles cénesthésiques. La négativité des examens complémentaires n'entrave en rien la conviction du sujet, qui peut réagir de façon hostile et agressive vis-à-vis des médecins, jugés incompétents et inefficaces [6].

L’évolution est chronique, avec des atténuations voire des rémissions temporaires sous traitement, qui fait estomper la lutte contre les parasites, alors que le délire reste souvent là, mais enkysté. Ce sont ces lésions cutanées qui représentent souvent le motif de consultation, paradoxalement non psychiatrique. Plusieurs auteurs, insistent sur l'aspect syndromique non spécifique de cette entité et sur le caractère non univoque de l’étiologie [11]. C'est ainsi qu'il peut se rencontrer dans de nombreux contextes pathologiques organiques et psychiatriques. Il s'agissait d'une dépression comorbide chez Mr AB.

### Sur le plan psychopathologique

Chez la personne âgée, la peau, cette enveloppe physique, assurant la cohérence et l'unité à l'individu, et élément capital du narcissisme, du contact et de la relation avec autrui, subit les modifications de la sénilité, tant au niveau apparent qu'au niveau fonctionnel. Ce qui représente chez le sujet âgé une perte d'objet. La perception du parasite peut représenter la récupération symbolique de l'objet perdu, la peau en l'occurrence [12]. Le vieillissement de la peau, rappelle souvent le rapprochement de la mort et risque d'alimenter une angoisse importante et profonde, vécue parfois sur un mode de morcellement. La vulnérabilité conséquente peut être exprimée de façon transposée, aboutissant au discours délirant [13]. D'un autre point de vue, ce parasite dévorant et donc dangereux peut représenter une punition implacable, un maléfice, qui doit conduire à la pourriture [2]. Le prurit et le grattage, ont été aussi interprétés, comme signe d'une culpabilité et d'un mécanisme punitif [3].

### Sur le plan thérapeutique

Le premier temps de la prise en charge est le plus souvent dermatologique, pour traiter les lésions cutanées. Parfois, un traitement antiparasitaire pourra être proposé pour faciliter la prise en charge psychiatrique [6]. D'autres disciplines peuvent être concernées, comme le médecin généraliste, le néphrologue, l'ophtalmologiste, le cas de Mr AB, et dont l'intervention débouchera par la suite à une collaboration psychiatrique. Un bilan organique général et dermatologique initial, permet d’éliminer une étiologie organique du prurit, qu'il faudra traiter spécifiquement. Une attitude empathique avec une écoute bienveillante, permet d’établir une relation de confiance avec le patient, facilitant son orientation ultérieure en psychiatrie. Le traitement psychiatrique est essentiellement basé sur la prescription des neuroleptiques ou des antipsychotiques atypiques qui sont mieux tolérés. Une coprescription d'antihistaminique peut permettre la réduction des démangeaisons, de l'anxiété, et une certaine sédation. Lorsqu'un état dépressif est repérable, ce qui est loin d’être rare, un antidépresseur pourra être associé. Enfin, un soutien psychothérapique est fondamental, permettant un accompagnement psychologique du patient [4]. L'explication de la maladie peut aider le patient à mieux intégrer les limites de son corps et à prendre conscience de la distinction entre Soi et non Soi.

## Conclusion

Le délire dermatozoïque, réputé comme rare, est probablement plus fréquent qu'on ne le décrit. Son expression somatique peut faire leurrer le clinicien, au prix d'une inefficacité du traitement symptomatique, et d'un coût important à la fois pour le patient, et pour le médecin. D'où l'importance d'un diagnostic positif précoce et d'une collaboration entre différentes disciplines concernées: psychiatrie, médecine générale, dermatologie, ophtalmologie, etc.

## References

[1] Anzieu (1995). Le moi-peau.

[2] Benattar B, Le Roux A, Van A, Hanon C, Pascal JC (2004). À propos du syndrome d'Ekbom. Ann Méd Psychol..

[3] Bourgeois M, Nguyen-lan A (1986). Syndrome d'Ekbom et délire d'infestation cutané - Revue de la littérature. Ann Méd Psychol..

[4] TISON P (2000). A propos d'un cas clinique de délire dermatozoïque d'Ekbom. La Revue de gériatrie.

[5] Pancrazi MP, Métais P (2003). Délires du sujet âgé Modalités thérapeutiques. Presse Med..

[6] Ait-Ameur A, Bern P, Firoloni MP, Menecier P (2000). Le délire de parasitose ou syndrome d'Ekbom. Revue Med Interne..

[7] Ekbom KA (1938). Der präsenile dermatozoenwahn. Acta Psychiatr Neurol Scand..

[8] Sylla A, Nadiaye M, Niang SO, Diémé B, Sylla O, Guèye M (2002). Le syndrome d'Ekbom ou délire dermatozoïque à propos de 5 cas à Dakar (Sénégal). Synapse..

[9] Trabert W (1995). 100 years of delusional parasitosis. Meta-analysis of 1223 case reports. Psychopathology..

[10] Lee WR (1983). Matchbox sign. Lancet..

[11] Even C, Goeb JL, Dardennes R (1997). Trouble affectif bipolaire et syndrome d'Ekbom: à propos d'un cas, L. L'encéphale..

[12] Faure C, Berchtold R, Ebtinger R (1957). Sur les parasitoses délirantes. L'évolution psychiatrique..

[13] Sizaret P, Simon JP (1976). Les délires à ectoparasite de l’âge avancé (syndrome d'Ekbom). L'encéphale..

[14] Bourgeois M, Rager P, Peyré F, Nguyen-lan A, Etchepare JJ (1986). Fréquence et aspects du syndrome d'Ekbom: enquête auprès des dermatologues français. Ann Méd Psychol..

[15] Aron-Brunetière R, Lôo H (1983). Psychiatrie et dermatologie. Encyclopédie Médico-Chirurgicale -Psychiatrie..

